# Characterization of the complete chloroplast genome of *Camellia granthamiana* (Theaceae), a Vulnerable species endemic to China

**DOI:** 10.1080/23802359.2018.1521310

**Published:** 2018-10-29

**Authors:** Weixi Li, Xianggang Shi, Wuxia Guo, Achyut Kumar Banerjee, Qun Zhang, Yelin Huang

**Affiliations:** a State Key Laboratory of Biocontrol and Guangdong Provincial Key Laboratory of Plant Resources, School of Life Sciences, Sun Yat-sen University, Guangzhou, Guangdong, China;; b Experiment Teaching Centre of Marine Sciences, School of Marine Sciences, Sun Yat-sen University, Zhuhai, Guangdong, China;; c Institute of Hydrobiology, Jinan University, Guangzhou, Guangdong, China

**Keywords:** *Camellia granthamiana*, Theaceae, complete chloroplast genome, Vulnerable species, phylogenomic analysis

## Abstract

*Camellia granthamiana* is a wild camellia resource endemic to China and is listed as a Vulnerable species globally. Here, we reported and characterized its complete chloroplast (cp) genome by using Illumina pair-end sequencing data. The total chloroplast genome size was 157,001 bp, including inverted repeats (IRs) of 26,042 bp, separated by a large single copy (LSC) and a small single copy (SSC) of 86,622 and 18,295 bp, respectively. A total of 131 genes, including 36 tRNA, 8 Rrna, and 87 protein-coding genes were identified. Phylogenetic analysis showed that *C. granthamiana* is sister to *C. sinensis* with 100% value support.

*Camellia*, a genus of about 250 species belonging to the family Theaceae, is notable for having species with economic (e.g. *C. sinensis*) and ornamental (e.g. *C. japonica*) purposes (Gao et al. 2005; Vijayan et al. 2009). *Camellia granthamiana* is a rare wild camellia resources endemic to China with a restricted distribution in the narrow regions of Guangdong province of mainland China and Hong Kong (Ming and Bartholomew 2007). Due to its multiple large and persistent bracteoles and sepals, the species is considered as a primitive taxa (Chang 1981). Furthermore, the plant is polyploid and can be used for cross-breeding, which makes them highly appreciated by gardeners. In recent years, habitat degradation and loss have resulted in the decrease of the population size of the species leading to its consideration as a Vulnerable species globally (IUCN: http://www.iucnredlist.org) and is under protection in Country Parks in Hong Kong now. In this context, a better insight into its genomics may contribute to our understanding of conservation of this species, and to achieve this objective, we assembled a complete chloroplast genome of *C. granthamiana*.

Total DNA was isolated from fresh leaves of an individual of *C. granthamiana*, collected from the field in the Sun Yat-sen University Botanic Garden. The source population of this plant comes from Hong Kong. The voucher specimen (Shixg 171204) was deposited in the Sun Yat-sen University Herbarium (SYS). Genome sequencing was performed on an Illumina Hiseq X Ten platform with paired-end reads of 150 bp. Total 6.79 Gb short sequence data were obtained, which was used to assemble the chloroplast genome in NOVOPlasty (Dierckxsens et al. 2017). The chloroplast atpB sequence of *E*ntandrophragma *excelsum* (GenBank accession number HQ158555) was used as the seed sequence. The genes in the chloroplast genome were annotated using the DOGMA program (Wyman et al. 2004). The circular chloroplast genome map was drawn using OGDRAW (Lohse et al. 2007).

The complete cp genome of *C. granthamiana* (GeneBank accession number MG782842) was 157,001 bp in size, containing a pair of inverted repeats (IRs) of 26,042 bp, which separated a large single copy region (LSC) of 86,622 bp and a small single copy region (SSC) of 18,295 bp. The cp genome contained 131 genes, including 87 protein-coding genes, 36 transfer RNA genes, and 8 ribosomal RNA genes. Most of the genes occurred as a single-copy in the LSC or SSC, while 19 genes had two copies in the IRs. The overall GC content of the cp genome was 37.3%.

To perform a phylogenomic analysis, 11 complete chloroplast genomes within the order of Ericales were considered along with *C. granthamiana*. One species from Ebenaceae (*Diospyros lotus*) was set as the outgroup species. The chloroplast genome sequences were aligned using MAFFT (Katoh and Standley 2013). Phylogenetic analysis using the maximum likelihood algorithm was conducted with RAxML (Stamatakis 2014) implemented in Geneious ver. 10.1 (http://www.geneious.com, Kearse et al. 2012). The result showed that all Theaceae species are clustered into a monophyletic group and *C. granthamiana* is sister to *C. sinensis* with 100% value support (Figure 1).

**Figure 1.**
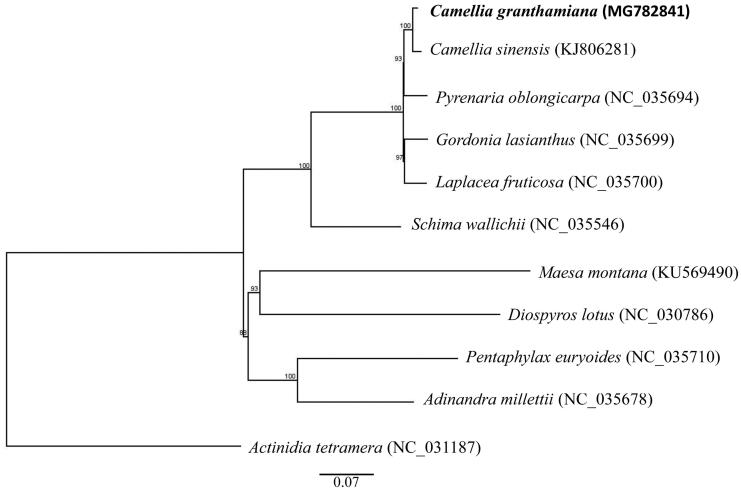
Maximum-likelihood tree based on the sequences of 11 complete chloroplast genomes. Numbers in the nodes are bootstrap support values from 1000 replicates. The position of Camellia granthamiana is shown in bold and GenBank accession numbers are listed behind each species name.

## References

[CIT0001] Chang HT 1981 A taxonomy of the genus *Camellia*. Acta Sci Nat Univ Sunyatseni, Monographic Series. 1:1–180.

[CIT0002] Dierckxsens N, Mardulyn P, Smits G 2017 NOVOPlasty: de novo assembly of organelle genomes from whole genome data. Nucleic Acids Res. 45:e18. 28204566 10.1093/nar/gkw955 PMC5389512

[CIT0003] Gao J, Parks CR, Du YQ 2005 Collected species of the genus Camellia and illustrated outline. Zhejiang: Zhejiang Science and Technology Press; p. 1–302.

[CIT0004] Katoh K, Standley DM 2013 MAFFT multiple sequence alignment software version 7: improvements in performance and usability. Mol Biol Evol. 30:772–780. 23329690 10.1093/molbev/mst010 PMC3603318

[CIT0005] Kearse M, Moir R, Wilson A, Stones-Havas S, Cheung M, Sturrock S, Buxton S, Cooper A, Markowitz S, Duran C, et al. 2012 Geneious Basic: an integrated and extendable desktop software platform for the organization and analysis of sequence data. Bioinformatics. 28:1647–1649. 22543367 10.1093/bioinformatics/bts199 PMC3371832

[CIT0006] Lohse M, Drechsel O, Bock R 2007 OrganellarGenomeDRAW (OGDRAW): A tool for the easy generation of high-quality custom graphical maps of plastid and mitochondrial genomes. Curr Genet. 52:267–274. 17957369 10.1007/s00294-007-0161-y

[CIT0007] Ming TL, Bartholomew B 2007 Theaceae In: Wu ZY and Raven PH, editors. Flora of China. Vol.12 St. Louis: Science Press, Beijing and Missouri Botanical Garden Press.

[CIT0008] Stamatakis A 2014 RAxML version 8: a tool for phylogenetic analysis and post-analysis of large phylogenies. Bioinformatics. 30:1312–1313. 24451623 10.1093/bioinformatics/btu033 PMC3998144

[CIT0009] Vijayan K, Zhang WJ, Tsou CH 2009 Molecular taxonomy of *Camellia* (Theaceae) inferred from nrITS sequences. Am J Bot. 96:1348–1360. 21628283 10.3732/ajb.0800205

[CIT0010] Wyman SK, Jansen RK, Boore JL 2004 Automatic annotation of organellar genomes with DOGMA. Bioinformatics. 20:3252–3255. 15180927 10.1093/bioinformatics/bth352

